# System and Method for Driver Drowsiness Detection Using Behavioral and Sensor-Based Physiological Measures

**DOI:** 10.3390/s23031292

**Published:** 2023-01-23

**Authors:** Jaspreet Singh Bajaj, Naveen Kumar, Rajesh Kumar Kaushal, H. L. Gururaj, Francesco Flammini, Rajesh Natarajan

**Affiliations:** 1 Chitkara University Institute of Engineering and Technology, Chitkara University, Punjab 140401, India; 2 Department of Information Technology, Manipal Institute of Technology Bengaluru, Manipal Academy of Higher Education, Manipal 576104, India; 3 IDSIA USI-SUPSI, University of Applied Sciences and Arts of Southern Switzerland, 6928 Manno, Switzerland; 4 Information Technology Department, University of Technology and Applied Sciences-Shinas, Shinas 324, Oman

**Keywords:** artificial intelligence, driver drowsiness, hybrid measures, MTCNN

## Abstract

The amount of road accidents caused by driver drowsiness is one of the world’s major challenges. These accidents lead to numerous fatal and non-fatal injuries which impose substantial financial strain on individuals and governments every year. As a result, it is critical to prevent catastrophic accidents and reduce the financial burden on society caused by driver drowsiness. The research community has primarily focused on two approaches to identify driver drowsiness during the last decade: intrusive and non-intrusive. The intrusive approach includes physiological measures, and the non-intrusive approach includes vehicle-based and behavioral measures. In an intrusive approach, sensors are used to detect driver drowsiness by placing them on the driver’s body, whereas in a non-intrusive approach, a camera is used for drowsiness detection by identifying yawning patterns, eyelid movement and head inclination. Noticeably, most research has been conducted in driver drowsiness detection methods using only single measures that failed to produce good outcomes. Furthermore, these measures were only functional in certain conditions. This paper proposes a model that combines the two approaches, non-intrusive and intrusive, to detect driver drowsiness. Behavioral measures as a non-intrusive approach and sensor-based physiological measures as an intrusive approach are combined to detect driver drowsiness. The proposed hybrid model uses AI-based Multi-Task Cascaded Convolutional Neural Networks (MTCNN) as a behavioral measure to recognize the driver’s facial features, and the Galvanic Skin Response (GSR) sensor as a physiological measure to collect the skin conductance of the driver that helps to increase the overall accuracy. Furthermore, the model’s efficacy has been computed in a simulated environment. The outcome shows that the proposed hybrid model is capable of identifying the transition from awake to a drowsy state in the driver in all conditions with the efficacy of 91%.

## 1. Introduction

Road accidents are one of the most severe transportation issues. As per the latest report by World Health Organization (WHO), 1.3 million people die from road accidents every year worldwide. Nearly fifty million people have suffered substantial fatal and non-fatal injuries, resulting in an immense monetary loss for drivers, victims and their families. The total cost borne by a country due to road accidents is approximately 3% of its gross domestic product [1]. According to the 2017 National Highway Traffic Safety Administration (NHTSA) data, 91,000 vehicle accidents were caused by driver drowsiness. The NHTSA also concedes that reported numbers are fairly low and that this count could be considerably higher [2]. Therefore, the research community has made significant contributions towards detecting driver drowsiness. Various measures have been investigated, such as subjective, vehicle-based, physiological and behavioral measures for driver drowsiness detection [3]. Each of these measures has some limitations, such as an inability to perform in all conditions. Thus, a robust Driver Drowsiness Detection System (DDDS) is required to detect the driver’s drowsiness at an early stage. Due to advancements in artificial intelligence-based technology, revolutionary ideas have been proposed to develop an efficient system that helps to detect driver drowsiness. A hybrid model can overcome such limitations by combining two or more measures for drowsiness detection in such a way that one method can reduce the limitations of others in order to improve the system’s overall accuracy [4].

This paper aims to propose a model for drowsiness detection using an amalgamation of behavioral and sensor-based physiological measures that will help to detect a drowsy state at an early stage. A detailed analysis of the hybrid model has been conducted in all conditions, i.e., low light, a face with eyeglasses or with a beard, to check the efficacy of the proposed model.

The rest of the paper is organized as follows: A state-of-the-art of the recent development in driver drowsiness detection systems is carried out in Section 2. Section 3 explains the methodology for detecting driver drowsiness. Section 4 explains the architecture of the proposed hybrid model. Section 5 discusses the implementation of the hybrid model and its results. The conclusion is presented in Section 6.

## 2. Background

### 2.1. Drowsiness

Drowsiness is the intermediate state between alertness and sleepiness. It is the biological state of a human being where the intensity of sleepiness is directly proportional to time. In a drowsy state, it is very difficult to keep one’s eyes open and to concentrate, as the head or the body is unstable. Frequently yawning is also one of the major signs of a drowsy state. Thus, driving in a drowsy state leads to vehicle accidents. Therefore, it is necessary to identify a drowsy state and to notify the driver early to avoid a collision. The drowsy state is very dangerous for the driver and road commuters [2,5]. Due to a drowsy state, it is very difficult for drivers to concentrate on the road while driving the vehicle, which restricts them from making quick decisions to control the vehicle components, i.e., brakes and steering [6].

Due to the circadian rhythm, most drivers become drowsy on national highways, notably late at night between 12:00 AM to 7:00 AM or in the afternoon between 2:00 PM to 4:00 PM [7]. Most of the time, the driver is driving alone on the highway and falls in the age group of 18 to 30. Previous studies have shown that the young generation is at high risk of a drowsy state, leading to fatal and non-fatal injuries [8]. Road accidents are unwanted things that happen quite often while travelling. Tracking road accident indicators like drunk driving, brake failure, bypassing traffic signals/rules and rash driving is much easier than accidents caused by the drowsy state of the driver. It is even more difficult to identify the primary cause of the accident due to a lack of a technical glitch in the vehicle or favorable road and weather conditions [3].

A smart affordable device is required to identify driver drowsiness at an early stage. A plethora of research has been done in the past to create a system that can identify driver drowsiness, but no system is ideal for driver drowsiness detection at an early stage. The present state of the art suggests that such dangers can be avoided by proposing an advanced AI-based system using an amalgamation of multiple measures.

### 2.2. Driver Drowsiness Detection Measures

There are two approaches, intrusive and non-intrusive, to identify drowsy driving. In an intrusive approach, the drowsy condition of a person is identified using physiological parameters, but in a non-intrusive approach, this is identified by installing devices and sensors on the vehicle. Based on intrusive and non-intrusive approaches, five different measures are utilized to identify driver drowsiness at an early stage [8,9,10]. These measures are as follows:

1. Subjective measures (SM)
2. Vehicle-based measures (VBM)
3. Physiological measures (PM)
4. Behavioral measures (BM)
5. Hybrid measures (HM)

**Figure 1:**
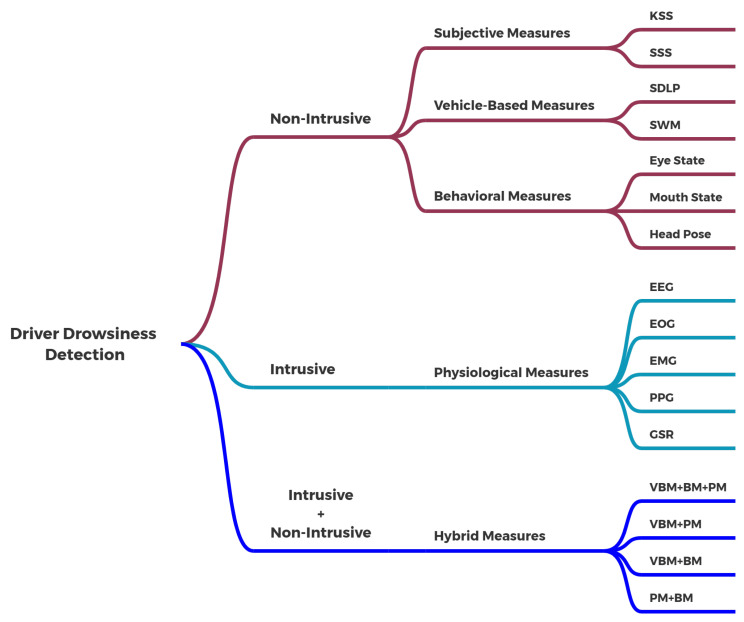
Driver drowsiness detection measures and respective techniques.

Physiological measures are considered intrusive, whereas subjective, vehicle-based and behavioral measures are considered non-intrusive. Hybrid measures are a combination of two or more measures. Figure 1 depicts the various measures for driver drowsiness detection and their respective techniques.

Subjective measures are used to detect driver drowsiness by gathering data from the driver in a simulated environment. The Stanford Sleeping Scale (SSS) and Karolinska Sleeping Scale (KSS) approaches help to gather the driver’s observations while operating the vehicle to assess the driver’s state. In SSS, seven levels of the Likert scale (1-feeling active to 7-extremely sleepy), and in KSS, nine levels of the Likert scale (1-extremely alert to 9-extremely sleepy) help to evaluate the different levels of drowsiness at a particular time. The drawback of a subjective measure is that it is impractical and produces biased results, making it impossible to utilize in real driving conditions [3].

By installing several types of sensors in various places in the vehicle, such as the driver’s seat and steering wheel, it is possible to apply vehicle-based measures. The two most common vehicle-based measures for driver drowsiness detection are Standard Deviation of Lane Positioning (SDLP) and Steering Wheel Movement (SWM). In SDLP, a camera is mounted on the front of the vehicle to track the lane position, which helps to identify the alert or drowsy state. Its biggest drawback is needing to rely on external variables like road markings, lighting and weather conditions. In SWM, various sensors positioned on the vehicle’s steering wheel gather information to aid in the detection of driver drowsiness. The primary issue associated with SWM is that it is expensive and has a high false positive detection rate, making it ineffective in real driving conditions [8].

**Figure 2:**
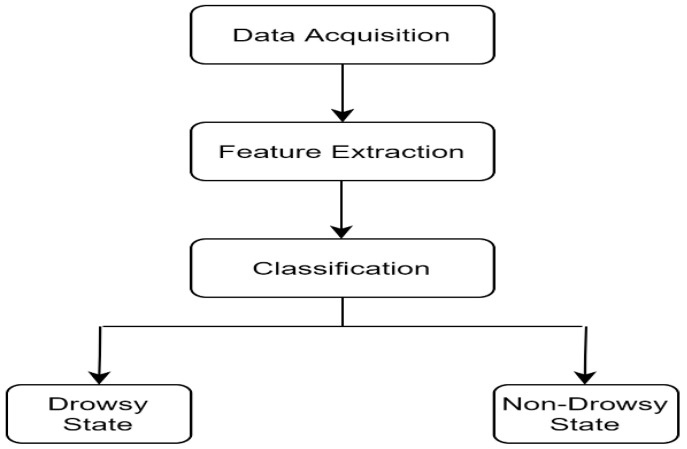
Detection of driver drowsiness using a GSR sensor.

Using physiological measures to identify driver drowsiness at an early stage provides promising results. A number of devices are directly attached to the driver to capture the relevant physiological parameters, such as electroencephalograms (EEG), electrocardiograms (ECG), electromyograms (EMG) and electrooculograms (EOG). Although physiological measures have a high level of accuracy, they are very intrusive. Using these highly intrusive devices in real driving conditions is challenging [3]. Therefore, small and lightweight physiologically based sensors that are less intrusive, such as a Galvanic Skin Response (GSR) sensor, can be used to record the physiological parameters [11]. A GSR sensor is a physiologically based sensor that is placed on the skin to capture the body’s skin conductance [12]. The literature suggests that detecting driver drowsiness using a GSR sensor is possible and can play a supportive role when the behavioral measures are ineffective. Figure 2 shows a flow diagram of the detection of the drowsy state of the driver using a GSR sensor.

The GSR sensor is first attached to the driver to collect bioelectric signals. Following data acquisition, it transmits the signals to the next phase for feature extraction. Based on the processed data, further raw data are converted into meaningful data by removing anomalies/missing frequencies and duplicate values. These data are transmitted again for classification after analysis. The binary classification technique efficiently classifies the drowsy or non-drowsy state.

Behavioral measures are based on the driver’s features, such as eyes, mouth and head inclination. To identify drowsy driving, the researcher primarily focuses on eye blink rate and percentage of eye closure (PERCLOS), which are further examined by machine learning (ML) and deep learning (DL) algorithms [13]. Other signs that can aid in detecting drowsiness in a driver include yawning and head movement. Due to their non-intrusive characteristics, these behavioral measurement techniques are frequently used in simulated and real driving conditions. The present state of the art reveals that behavioral measures are more accurate than vehicle-based measures [14].

**Figure 3:**
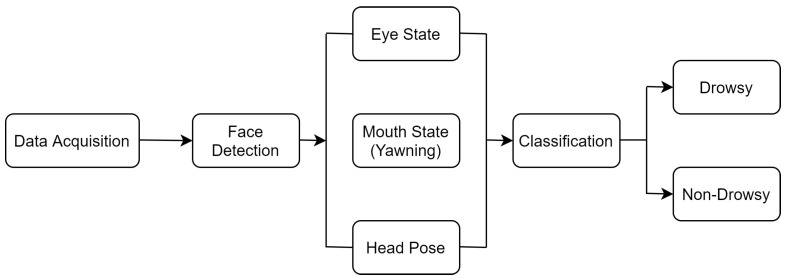
Detection of driver drowsiness using behavioral measures.

Due to its non-intrusiveness, the behavioral measure is one of the most widely used drowsiness detection techniques [15]. A camera is mounted on the dashboard of the vehicle to capture the driver’s facial features [14]. Figure 3 depicts the procedure to determine the drowsy state of the driver using behavioral measures. Three phases comprise the entire process: data acquisition, feature extraction and classification. The first step in data acquisition is gathering the driver’s image or video. Thereafter, in the second phase, the face is detected by applying pre-processing techniques. Through the feature extraction phase, the region of interest (ROI) is identified. The ROI includes capturing eye, mouth and head pose using machine or deep learning-based algorithms. In the classification phase, the binary classification method is used to evaluate the drowsy or non-drowsy state of the driver.

**Figure 4:**
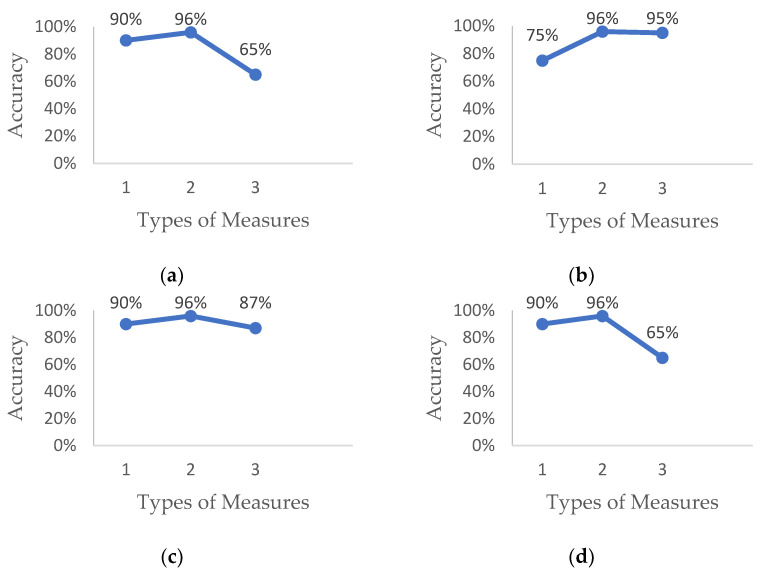
Accuracy of driver drowsiness detection on the basis of different conditions: (**a**) low lighting conditions; (**b**) road conditions; (**c**) driver with beard; (**d**) driver wearing eyeglasses. (1) Vehicle-Based Measure (2) Physiological Measure (3) Behavioral measure.

Three driver drowsiness detection measures have been compared under four different conditions, i.e., poor illumination environments, road conditions, drivers wearing eyeglasses and drivers with beards or moustaches, to find the most effective methods to detect driver drowsiness. Figure 4 shows the comparison and indicates that the physiological measure shows better results among all other measures in all conditions, but it is highly intrusive [4].

Behavioral measures also show promising results for driver drowsiness detection with higher accuracy in normal conditions, but the accuracy decreases drastically in certain conditions such as low light and drivers with eyeglasses. In addition, the non-reliable secondary dataset used by artificial intelligence-based algorithms is also the root cause of a false positive driver drowsiness detection rate [10]. Due to these limitations, it is not possible to use this measure alone in real driving conditions.

The limitations of individual measures can be overcome by combining two or more measures in such a way that one technique can reduce the limitations of the others to improve the system’s overall accuracy [16]. Table 1 reveals the possibility of various hybrid approaches used for driver drowsiness detection. Hybrid measures combine two or more measures that help to develop a highly accurate and reliable driver drowsiness detection system. The majority of the research on hybrid measures has only been done in a simulated environment. Due to their high cost and difficulty of implementation in actual driving situations, all measures cannot be applied at once [4]. Due to their dependence on the state of roads and lane markings, vehicle-based measures, when combined with other measures, produce a significant false positive detection rate. Therefore, combining vehicle-based measures with other measures in real driving conditions is challenging.

Among all possible combinations, the behavioral and physiological measures produce promising results. However, the physiological measures are extremely intrusive and thus challenging for the driver to wear them and drive the vehicle. Numerous studies have confirmed that biological sensors can be replaced with intrusive physiological components to identify driver drowsiness. Due to technological advancements in the area of AI and biological sensors, hybrid measures can be utilized for driver drowsiness detection at an early stage.

## 3. Materials and Methods

Several approaches to developing a model for detecting driver drowsiness that can be used effectively have been investigated. To identify the most effective method and review the current advancements in the area of DDDS, sixty-eight research publications from various sources, including IEEE, Google Scholar, ScienceDirect and ResearchGate, have been selected. Thirty-one papers have been shortlisted out of sixty-eight studies that discuss face detection techniques, hybrid measures and deep learning algorithms.

**Figure 5:**
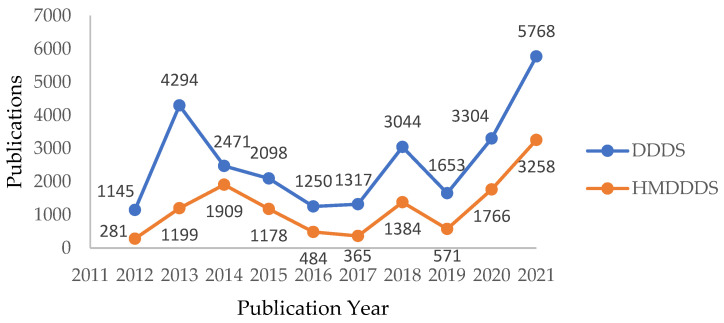
Publication trends based on DDDS and hybrid-based DDDS from 2012 to 2021.

A total of 26,344 articles have been published that help the research community to build an efficient driver drowsiness detection system, of which 12,395 articles are based on hybrid models, e.g., DDDS. Figure 5 shows the publication trends from 2012 to 2021 in DDDS and hybrid model-based driver drowsiness detection systems (HMDDDS). These publication trends reveal that the research community has shown intense interest in building an efficient DDDS to reduce accidents and protect people’s precious lives [21].

**Figure 6:**
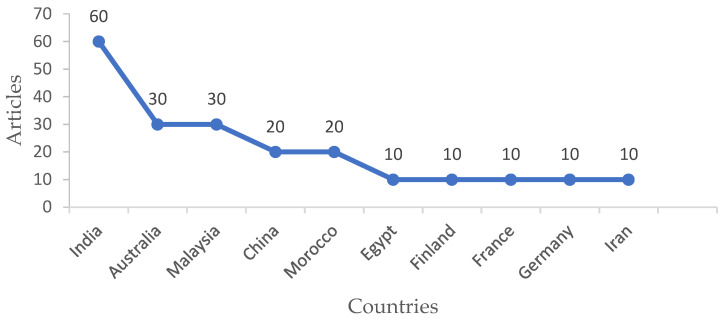
Frequency of publications based on DDDS by country.

Figure 6 shows that developing countries like India are more interested in developing a driver drowsiness detection system. The number of publications in India is almost double that of other countries.

### 3.1. Hardware Requirements

For implementation purposes, the following hardware components are used:

- Raspberry Pi 3 B+
- Pi Camera v2 8 MP
- GSR Sensor
- Analog-to-digital converter

Table 2 shows the specification of all hardware components used to develop the hybrid model. The Raspberry Pi 3 Model B+ features a 64-bit quad-core processor running at 1.4 GHz, dual-band wireless LAN and Bluetooth 4.2/BLE. This specification makes it easier to construct a microcontroller-based driver drowsiness detection system [22].

**Figure 7:**
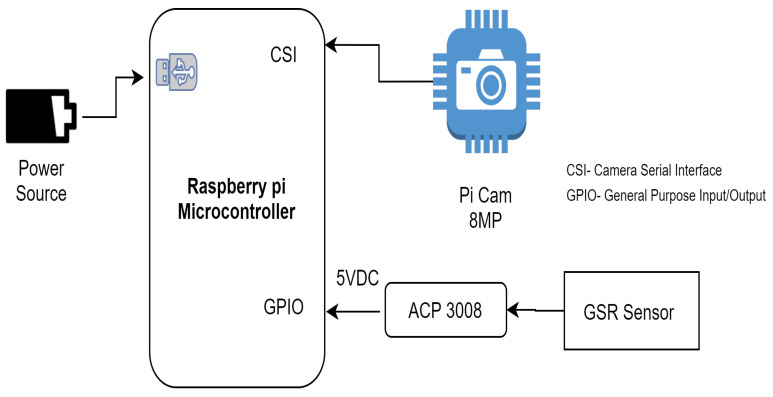
Schematic diagram of the hardware implementation of the proposed model.

**Figure 8:**
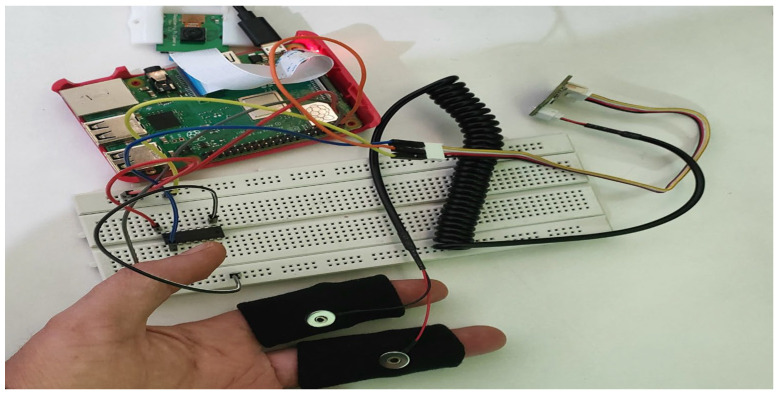
Raspberry Pi 3 B+ microcontroller with camera and sensor.

The Raspberry Pi Camera v2 is a high-quality image sensor for the Raspberry Pi microcontroller. It can capture high-quality images and videos that help to detect the driver’s facial features [23]. It connects to the Raspberry Pi via the CSI interfaces on top of the board. The Grove GSR Sensor is used in the proposed model to collect the electrical conductance of the skin by attaching two electrodes to any of the two fingers on one hand of the driver. The GSR Sensor sends a small amount of electrical current through one electrode and measures the intensity of the current received on the other. Skin conductance (SC) fluctuates with skin moisture. The body’s reaction to physical exertion and stress can be assessed using the skin conductance of the person [24]. Due to the non-availability of analog inputs in the Raspberry Pi 3 model B+, a MCB3008 Integrated Circuit (IC) is used as an analog-to-digital converter. The Python code is burnt on the Raspberry Pi to communicate with the Pi camera and GSR sensor to capture images and skin conductance, respectively. The complete hardware implementation is demonstrated by the schematic diagram in Figure 7 and Figure 8, showing the Raspberry Pi 3 model B+ connected with Raspberry Pi camera and GSR sensor using MCP3008 IC.

**Figure 9:**
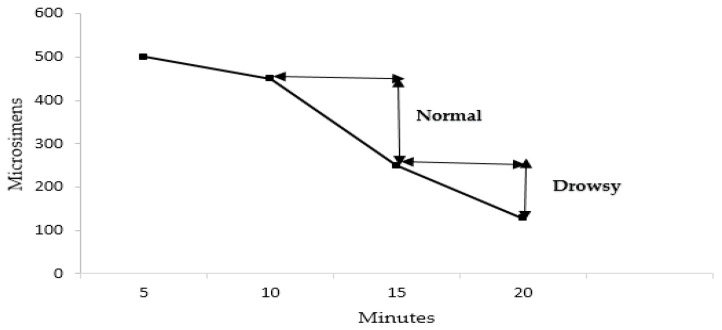
GSR skin conductance value.

During drowsiness, it is observed that the GSR value tends to decrease. The conductivity of the skin is used to calculate the GSR value. The equation to measure skin response is:

C = (1/R)(1)

where C represents the skin conductance, which is inversely proportional to resistance (R). The skin conductance can be measured in microsiemens (µs), where the normal range lies between 250 µs to 450 µs for a normal person. The GSR value, often between 128 µs and 250 µs, indicates the driver’s drowsy state, which further helps to identify the driver’s state, i.e., normal or drowsy, as depicted in Figure 9. It is determined by calculating the slope of the GSR, which provides the average rate of absolute change from a group of data observed over a period of time. It is determined by averaging the initial difference of the skin conductance signal’s absolute value [25].

To analyze the GSR signal, four parameters are used: mean, standard deviation, kurtosis and skewness. The baseline of the signal is its mean. The standard deviation reveals modifications to the signal’s baseline. The signal’s flatness concerning the normal distribution is assessed using kurtosis. A positive number for kurtosis often denotes a signal that is leptokurtic, which is flatter than the normal distribution, whereas a negative value denotes a signal that is platykurtic, which is less flat than the normal distribution [26]. The implemented equation for kurtosis is the following:

(2) $$Kurtosis\left(x_{1}\ldots x_{n}\right)=\left\{\frac{1}{n}\overset{n}{\underset{j=1}{\sum }}{ [\frac{x_{j}-\bar{x}}{\sigma }]}^{4}\right\}$$

where *x* is a random variable having *n* observations. The above kurtosis equation calculates the sum of deviation from the mean value divided by the standard deviation power of 4. The term in the equation describes the shape of the GSR signal, whether tall or flat.

*Skewness* is shown to indicate the GSR signal’s symmetry with respect to its baseline. A positive skewness number would represent a rightward skew in the signal, whereas a negative skewness value would represent a leftward skew. The implemented equation for skewness is as follows:

(3) $$Skewness\left(x_{1}\ldots x_{n}\right)=\frac{1}{n}\overset{n}{\underset{j=1}{\sum }}{ [\frac{x_{j}-\bar{x}}{\sigma }]}^{3}$$

where *x* is the random variable having *n* observations. The above skewness equation is the sum of the deviation from the mean value divided by the standard deviation power of 3. The term used in the equation describes the symmetry of the GSR signal, whether it is a positive skew distribution or negative skew distribution. All these equations are utilized in the proposed hybrid model to calculate efficacy, as equations will remain same whether it is a hybrid model or a model with a single measure.

Various tools and techniques have been thoroughly addressed in the preparation of this work. The detailed analysis of different face detection techniques and feature extraction using AI-based algorithms are explained below, followed by the hybrid model.

### 3.2. Face Detection Techniques

Face detection is the crucial step in detecting the face of the driver and extracting the facial features to evaluate the drowsy state of the driver [27,28,29]. The three most popular techniques for detecting the face below are available below to implement behavioral measures.

1. OpenCV Haar Cascade
2. Davis King library (Dlib)
3. Multi-task Cascaded Convolutional Neural Network (MTCNN)

Face detection is important for both identifying faces and extracting facial features. Many face detection methods can capture, recognize and process a face in real time while extracting various facial features. In the present era, many electronic devices include built-in facial detection software that may verify the user’s identification. This section explains all face detection techniques that help to select the appropriate technique for the proposed hybrid model.

The OpenCV Haar Cascade classifier is an effective method to detect objects. Paul Viola and Michael Jones first suggested the strategy in 2001 [30]. The face coordinates can be obtained using a mathematical model to determine the integral image. It is calculated as:

(4) $$I_{i}\left(x,y\right)=\overset{x.y}{\underset{i=1}{\sum }}i\left(x^{\prime },y^{\prime }\right)$$

where (*x*, *y*), (*x*’, *y*’), the brightness of the pixel in the image according to the coordinates, and *I_i_* (*x*, *y*) the value of the *i*th element of the integral image with coordinates (*x*’, *y*’). The integral image is used to quickly determine the brightness of certain areas of the image, regardless of the size or location of the image. OpenCV is an ML-based open-source computer vision library with a trainer and a detector. To detect a human face and eye, a pre-trained classifier can be used as an XML file [31]. This technique quickly detects the object efficiently, but it cannot detect faces under occlusion. It also generates many false predictions during the face detection procedure [27].

Dlib is another open-source library used for implementing various machine-learning algorithms. It helps to detect human facial features in images and videos. The Dlib library provides two algorithms that can be used for face detection, which are Histogram of Oriented Gradients (HOG) + Linear Support Vector Machine (SVM) and CNN based. Dlib HOG is a simple yet powerful tool to detect the face and is widely used [28]. It works with the combination of an SVM machine-learning algorithm that effectively detects the faces of the person. It is a fast and lightweight model that works without special hardware requirements. However, the highly accurate Dlib CNN face detection method is employed for face detection. CNN is a deep learning-based algorithm with Dlib that helps detect the face from different angles. Dlib is more accurate than OpenCV Haar Cascade, but the computational cost is very high and unable to run in real-time conditions [27].

One of the most popular and reliable facial recognition methods used today is MTCNN. It is a multi-task cascaded convolutional neural network that uses images to detect the face and facial features. It is a deep learning algorithm to identify faces and facial features accurately [29]. The whole concept of MTCNN can be explained in three stages, as mentioned below:

P-NET: MTCNN generates several frames that successfully scan the entire image from the top left corner to the bottom right corner.

R-NET: Most frames without faces are rejected by the next layer of CNN, which uses the data from P-Net as input.

O-NET: The results of this step are more detailed than that of R-Net. The facial landmark position is the output of this stage after detecting a face from the given image/video.

Identifying prominent facial features is known as facial landmark detection, which aids in monitoring the driver’s drowsiness. MTCNN allows for the detection of five facial landmarks: two for the eyes, one for the nose and two for the mouth. By using these landmarks, drowsy detection can be identified by efficiently using these landmarks. Apart from this, facial landmarks can be effectively used for behavior detection while online [32].

Table 3 shows the comparative analysis of various face detection techniques. Five parameters are used to analyze the best technique for driver drowsiness detection [27]. Table 3 revealed that the MTCNN provides a better result than the OpenCV Haar Cascade and Dlib. MTCNN can also detect the sides of faces from the images [29]. The only limitation recorded in MTCNN is that it takes more time to train the system than other techniques. From this analysis, it is concluded that MTCNN is the best technique for face detection implementation.

## 4. Architecture of Hybrid Model

**Figure 10:**
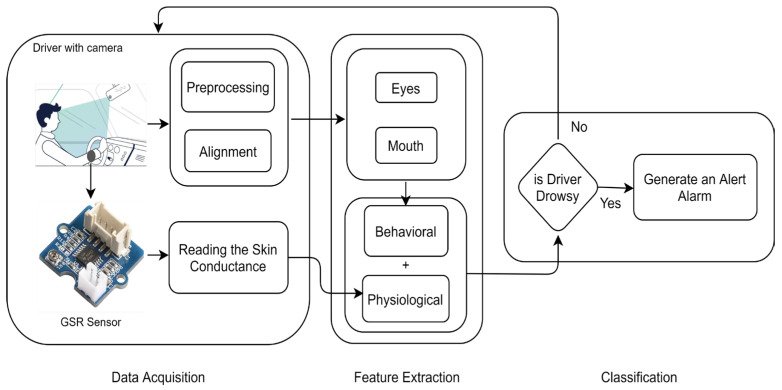
The architecture of the proposed hybrid model.

The proposed hybrid model is the amalgamation of two measures: behavioral and physiological. Figure 10 depicts the architecture of the hybrid model. This model has been divided into three phases.

1. Data Acquisition
2. Feature Extraction
3. Classification

Data Acquisition: Data collection is an essential first phase for the driver’s drowsiness detection. Video data from the pi camera is collected to identify the driver’s face. A camera is mounted on the vehicle’s dashboard to record the driver’s face. Subsequently, the captured video is divided into multiple images. The face is detected using a trained dataset provided by National Tsing Hua University (NTHU) [33]. A physiologically based GSR sensor is attached to the driver’s fingertips to collect the driver’s skin conductance. The GSR sensor and the pi camera are connected to the Raspberry-Pi controller, which detects the driver’s face and skin conductance in real time. The driver’s bioelectrical signals are then passed to the next phase, which is utilized to detect driver drowsiness.

Feature Extraction: In the second phase, after data acquisition, the data are processed for feature extraction of the face, which is accomplished using the MTCNN algorithm. MTCNN helps to identify the face in the image and apply landmarks from the data [29]. This information will be used subsequently to classify the driver’s drowsiness. The raw data extracted by the GSR sensor are converted into digital form and further transmitted for classification. Two parameters are employed for eye detection and mouth detection utilizing PERCLOS and Frequency of Mouth (FOM), respectively, to detect the drowsy state of the driver using behavioral measures. PERCLOS, a drowsy analysis technique, displays the ratio of closed eyes based on the frequency of open and closed eyes. It is calculated as:

PERCLOS = NC/NOCL × 100(5)

At any given time, NOCL stands for the total number of closed and open eye frames, whereas NC stands for closed eye frames. FOM is the ratio of open squares to all squares over a specified period. FOM calculations resemble PERCLOS calculations.

FOM = NOM/NMCO × 100(6)

NOM represents the number of open-mouth frames, and NMCO represents the total number of closed and open-mouth frames in a specified period [34].

Classification: In the third phase, the behavioral and physiological measures are integrated to classify the driver drowsiness detection system. Various types of ML- and DL-based classifiers can be integrated with the algorithm to evaluate the drowsy state of the driver. SVM, CNN and HOG classifiers have been widely used for the evaluation [35]. A classification method like SVM is used to evaluate the driver’s current condition. If the classifier determines that the user is not in a drowsy condition, the process begins at the beginning. If the classifier detects a drowsy state, then it generates an alarm to inform the driver or it goes back to the first phase and restarts the procedure. The steps of the proposed hybrid model are mentioned in the Algorithm 1.
**Algorithm 1**: Working of Hybrid Model1:Mount a camera on the dashboard of the vehicle and attach a GSR sensor on the fingers of the driver.2:Capture the image and collect the reading of the skin conductance of the driver.3:Detect the face in the captured images and forward the images to step 4 for feature extraction.4:Extract facial features like the eyes and mouth from the images using MTCNN and convert the analog reading of skin conductance into digital form. 5:Forward the above results to step 6 for classification.6:Classify the current state of the driver and forward the result to next step.7:Generate an alarm if the driver is drowsy or restart the procedure at step 2.

## 5. Results and Discussion

**Figure 11:**
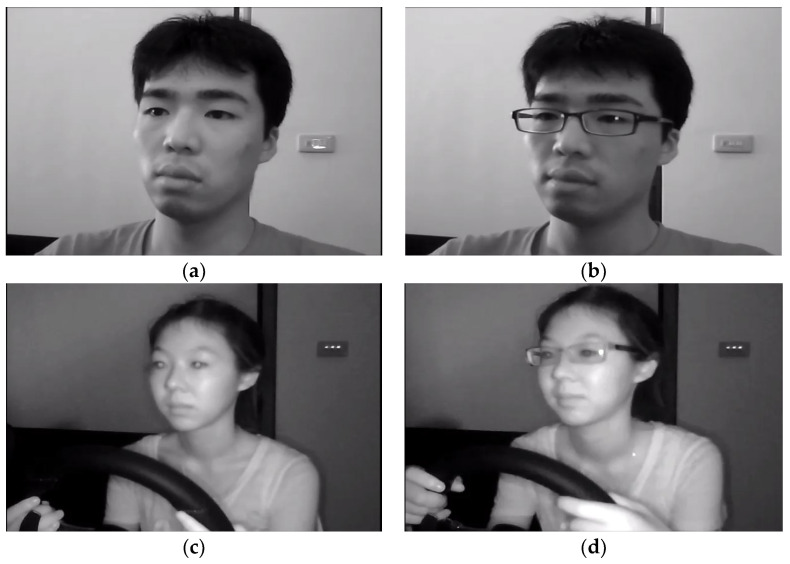
NTHU dataset with different states with glasses and without glasses: (**a**,**c**) NDHU-DDD sample without glasses; (**b**,**d**) NDHU-DDD sample with glasses [33].

Researchers frequently use the NTHU secondary dataset for driver drowsiness detection [33]. The NTHU-DDD dataset consists of 36 people of various ethnicities who were seen yawning, blinking slowly, dozing off and wearing glasses or sunglasses under both day and night lighting conditions. The sample videos of male and female drivers from various ethnic groups comprise the NTHU-DDD dataset. The sample video consists of different types of drowsy and non-drowsy events. These videos were also recorded in different lighting conditions [34,36]. The overall accuracy of this dataset is higher than the other datasets available for training the driver drowsiness system [37]. Some samples of the dataset images are shown in Figure 11.

**Figure 12:**
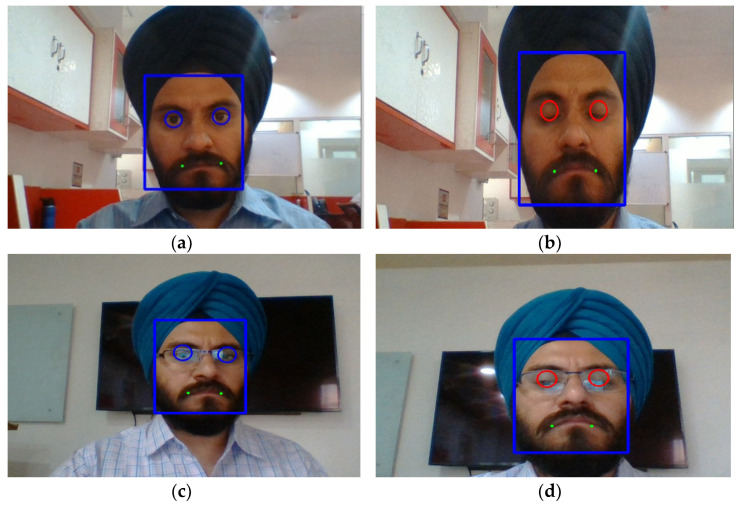
Detecting facial landmarks using MTCNN: (**a**,**b**) face with open and closed eyes in normal conditions; (**c**,**d**) face with open and closed eyes with glasses.

During the training process, the datasets in the study are divided into three groups: a training set, a validation set, and a test set. The dataset contains a variety of NTHU-DDD used in the multi-task architecture, since the mouth and the eyes are both identified on images concurrently from the dataset. The NTHU-DDD dataset and MTCNN are utilized together to detect the drowsy state. An image pyramid is created after scaling the source image for input. After that, the altered image is sent to the P-net, which creates numerous candidate face windows of various sizes. This process produces a somewhat unpolished output. The R-net is then used to filter out even more overlapping windows and discard them. Finally, the O-net determines whether the candidate window should remain open or closed. In the end, the main facial feature points are revealed. Four landmark points in the facial region are located and recorded using the MTCNN algorithm. To locate the driver’s mouth and eye areas in the video, PERCLOS and FOM are calculated. The mouth and eye detection regions are displayed in Figure 12. When the user closes his eyes, the area of the eye turns red from blue. This algorithm also detects the user’s eyes opening and closing status, even with glasses.

For driver drowsiness detection, a combination of behavioral and sensor-based physiological measures was deployed on eight individuals between 25 and 48 years of age. This experiment was carried out in a simulated environment using simulation parameters like PERCLOS, FOM and skin conductance. PERCLOS and FOM values were used in conjunction with skin conductance to determine a person’s current state, such as alertness or drowsiness. When a driver is drowsy, behavioral reactions such as eye state, yawning, and biological signals continue to vary. The drowsy state of the driver can thus be assessed by calculating the PERCLOS, FOM and skin conductance. When the driver is driving normally, the duration of the eyes remaining open is much longer than the time of closure, so the PERCLOS values are below the threshold (0.24). When the driver is drowsy, the duration of closure is longer than the time of opening. Similarly, when a person yawns, the mouth remains open for a few seconds (6 s). The driver is considered to be drowsy when PERCLOS > 0.24, FOM > 0.16 and SC < 250. When the PERCLOS > 0.24, FOM > 0.16 and SC > 250, the person is less sleepy, and PERCLOS < 0.24, FOM > 0.16 and SC > 250 show the normal state of the driver. Table 4 represents the mean values of PERCLOS, FOM and skin conductance for the eight subjects. MTCNN is used for detecting the facial features. The accuracy of the model is achieved by training the model (split ratio), where 70% from the provided dataset is used for training and 30% is used for testing purposes, which helps to achieve 91% accuracy by reducing the false positive rate. The accuracy of the model is achieved by:

$$\mathrm{Accuracy}=\frac{\mathrm{TP}+\mathrm{TN}}{\mathrm{TP}+\mathrm{TN}+\mathrm{FP}+\mathrm{FN}}$$

False Positive (FP): Subject misclassified as “drowsy,” where the subject was actually normal.

False Negative (FN): Subject misclassified as “normal,” where the subject was actually drowsy.

True Positive (TP): Subject truly classified as “drowsy,” where the subject was drowsy.

True Negative (TN): Subject truly classified as “normal,” where the subject was normal.

The pi camera is mounted in front of the driver to record facial features, and the GSR sensor is attached to the driver’s fingers to collect the skin conductance data. This test was conducted on eight individuals and completed in two weeks. Due to the availability of just one simulated driving system, few users were available for testing. An experiment was conducted in the morning on security guards who were assigned the night shift on previous day to determine the true value of the drowsiness scale.

Table 5 displays the GSR levels for the eight individuals. It displays each person’s skin conductance while driving the vehicle in a simulated environment. The value shows how stress or drowsiness affects a person’s present state when driving over time. Throughout the experiment, the system uses the sensor to track the subject’s GSR values and displays the skin’s response every second. Each person’s skin response evolves gradually over time. The skin response levels change marginally for the first several minutes while the drivers remain active. However, the GSR value shows a change in extremely low dermal activity with the passage of the 15-minute mark, which may cause drowsiness. Out of the eight individuals, the GSR value of Subject 3 is observed as the lowest value (74.22 µs) and the GSR value of Subject 2 as the highest value (348.2 µs).

**Figure 13:**
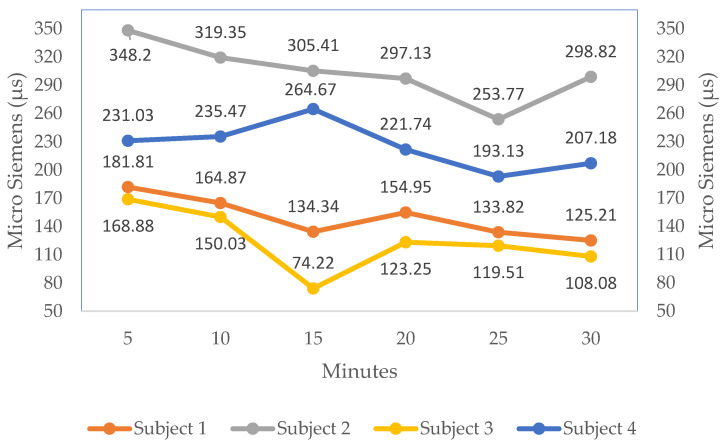
Skin conductance graph of the first four subjects.

**Figure 14:**
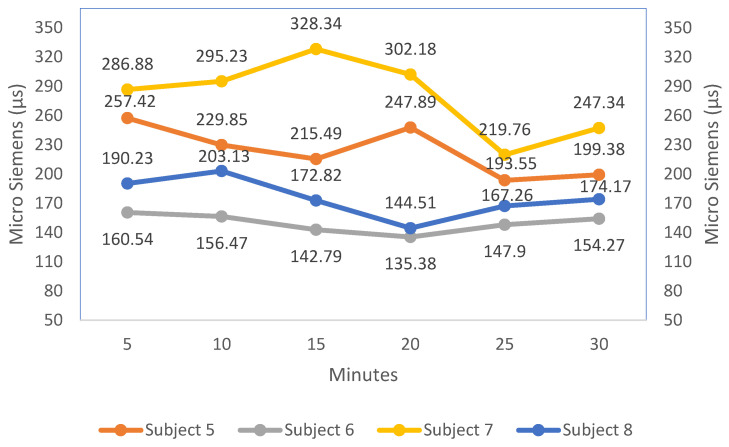
Skin conductance graph of the last four subjects.

The line graph depicts the variation in galvanic skin response for the eight individuals over time (every 5 minutes) in Figure 13 and Figure 14. The line chart shows the change in the GSR value of all the individuals. The GSR value of Subjects 1, 3, 5, 6 and 8 falls in the range of the drowsy state, which is explained in Figure 9. The lower GSR value of the subject indicates the drowsy state of the driver. Hence, detecting drowsiness using GSR values reduces the limitations in a situation where the camera is not working effectively.

The parametric measures of the proposed hybrid model are compared with the other driver drowsiness detection measures proposed by other researchers. Table 6 compares the state-of-the-art studies with the proposed model [3,4,8,9,38]. The subjective measures are evaluated according to the KSS and SSS ratings. These ratings cannot provide parametric measures due to self-introspection that alerts the driver. In vehicle-based measures, 88% accuracy is provided, but it did not work without road markings or in low light conditions. The physiological measures provide promising parametric measures, but they are highly intrusive. It is difficult for the driver to use such intrusive components in real driving conditions. The behavioral measures outperformed with respect to all other measures, but it is not feasible to use them alone due to the high false positive detection rate and inability to work in low lighting conditions. In sensor-based physiological measures, the drowsy state was identified by evaluating the respiration rate of the driver using the impulse radio ultra-wideband (IR-UWB) radar system [38]. The accuracy of the system claimed by the researcher is 86%, but it lacks consistency in data collection. It depicts that the proposed hybrid model outperforms in all above-stated conditions. It uses a camera and GSR sensor that simultaneously collect the various behavioral measures, such as eye and mouth position, and sensor-based physiological measures, such as the driver’s skin conductance level. The proposed model has a few limitations that researchers intend to overcome in the future and is outlined as follows: the model’s outcome is analyzed using the single secondary video-based dataset; a large dataset is required for further investigation. Limited drivers of a certain age were considered during the data gathering; more individuals can be considered. The GSR sensor was connected to the microcontroller using wires that may disturb the driver while driving; the GSR sensor needed to send the readings wirelessly to the microcontroller, which helped the driver to drive the vehicle easily. In addition, advanced deep-learning techniques can help to build a real-time driver drowsiness detection system [39].

## 6. Conclusions

Drowsiness detection is vital to save precious human life and monetary losses. This study proposes a hybrid drowsiness detection model using multiple measures to detect driver drowsiness in all conditions that also reduces the false positive rate. It has been concluded that none of the four distinct measures, taken separately, can ensure accuracy. Each measure has limitations in different contexts and is ineffective in detecting drowsiness. These limitations can be eliminated by combining two or more measures to detect driver drowsiness and making the system work under all conditions. The literature review indicates that combining behavioral measures, which are non-intrusive, with sensor-based physiological measures, which are intrusive, produces better results and overcomes certain limitations. A hybrid model that helps to detect driver drowsiness in all conditions is proposed. The driver’s facial features are extracted using a camera as a behavioral measure and the GSR sensor as physiological measure to investigate the transition from alert to drowsy state. Improved accuracy and reduced false positive detection rates are the outcome of the proposed model. This model considers a driver to be drowsy when PERCLOS > 0.24, FOM > 0.16 and SC < 250. When PERCLOS > 0.24, FOM > 0.16 and SC > 250, the person is less sleepy, and PERCLOS < 0.24, FOM > 0.16 and SC > 250 shows the normal state of the driver. The mean value of PERCLOS and FOM are utilized in conjunction with skin conductance to identify the driver’s current condition. Results are compared with the threshold values of PERCLOS, FOM and skin conductance of the body, e.g., 0.24, 0.16 and 250, respectively. Additionally, the proposed hybrid model is also cost-effective and easy to implement. The efficacy of the proposed model may be improved by integrating other sensors, such as the PPG, pulse rate sensor and IR-UWB radar. In addition, advanced deep-learning techniques can build a true real-time driver drowsiness detection system.

## Figures and Tables

**Table 1:** Possibility of combination of various measures.

Ref.	Hybrid Measures	Accuracy	Advantage	Limitation
[17]	Behavioral + Vehicle-based	91%	Ease of use	High false positive detection rate and dependent on geographical conditions
[18]	Behavioral + Physiological	98%	High accuracy	Highly intrusive
[19]	Vehicle-based + Physiological	93%	High accuracy	Extremely intrusive and geographically dependent
[20]	Vehicle-based + Physiological + Behavioral	81%	High accuracy and ease of use	Expensive and more challenging to implement in real driving conditions

**Table 2:** Hardware specifications.

Name of Component	Specifications
Raspberry Pi 3 B+	64-bit quad-core processor running at 1.4 GHz, dual-band 2.4 GHz and 5 GHz wireless LAN, and Bluetooth 4.2/BLE
Pi Camera v2	8 Mega Pixel
GSR Sensor	V2.0, 3.3/5 VDC
Analog-to-digital converter	MCP3008

**Table 3:** Comparative analysis of various face detection techniques based on various parameters.

Parameters	Face Detection Techniques		
	OpenCV Haar Cascade	DLIB	MTCNN
Work in real-time conditions	✓	✓	✓
High accuracy in different conditions	✓	✓	✓
High efficiency	✕	✕	✓
Detect the sides of faces	✕	✕	✓
Less time for training	✓	✓	✕

**Table 4:** Mean value of PERCLOS, FOM and skin conductance for eight subjects.

	Subjects	S1	S2	S3	S4	S5	S6	S7	S8
Parameters									
PERCLOS		0.37	0.21	0.42	0.26	0.22	0.36	0.18	0.32
FOM		0.23	0.11	0.21	0.14	0.17	0.22	0.09	0.13
Skin Conductance		162.5	275.4	128.9	225.5	247.3	166.2	274.9	178.7

**Table 5:** Representation of skin conductance of the eight subjects.

Duration	S1	S2	S3	S4	S5	S6	S7	S8
5	181.81	348.2	168.88	231.03	257.42	160.54	286.88	190.23
10	164.87	319.35	150.03	235.47	229.85	156.47	295.23	203.13
15	134.34	305.41	74.22	264.67	215.49	142.79	328.34	172.82
20	154.95	297.13	123.25	221.74	247.89	135.38	302.18	144.51
25	133.82	253.77	119.51	193.13	193.55	147.9	219.76	167.26
30	125.21	298.82	108.08	207.18	199.38	154.27	247.34	174.17

The eight subjects are labeled S1–S8.

**Table 6:** Comparison proposed for hybrid model with different studies.

Reference	Measures	Drowsiness Detection Methods/Sensors	Accuracy	Limitations
[3]	Subjective	KSS and SSS	NA	Cannot be used in real driving conditions
[8]	Vehicle-Based	SWM and SDLP	88%	High false positive detection rate
[9]	Behavioral	PERCLOS and Yawning	Close to 100%	Does not work in all conditions
[4]	Physiological	EEG and EMG	97–99%	Highly intrusive and cannot be used in real driving conditions
[38]	Sensor-Based Physiological	Respiration Rate	86%	Low accuracy
Proposed Hybrid Model	Behavioral + Sensor-Based Physiological	PERCLOS, Yawning and Skin Conductance	91%	Need more investigation using large number of individuals in real driving conditions

## Data Availability

The National Tsing Hua University (NTHU) dataset is used with prior permission.
